# 

**Published:** 1817-07

**Authors:** 


### JOURNAL AND REVIEW.

CONTAINING,

I. Select Original Communications.

II. Comprehensive Analytical Review of all important Medical Publications; presenting a fair Epitome of the Medical Literature of the Day.

III. Select Translations from Foreign Medical Journals.

IV. Medical Miscellanies, Foreign and Domestic; Anonymous Communications, &c. &c.

CONDUCTED BY

WILLIAM SHEARMAN, M. D.

Licentiate of the Royal College of Physicians; Physician to the London Dispensary; and to the Universal Dispensary for Children;

JAMES JOHNSON, M. D.

Surgeon to His Royal Highness the Duke of Clarence's Household; Author of "The Influence of Tropical Climates on European Constitutions;" and of "The Influence of the Atmosphere on the Health and Functions of the Human Frame, &c.;

AND

SHIRLEY PALMER, M. D.

Of the University of Glasgow.

OMNIA VINCIT LABOR SI LABOR IPSE VOLUPTAS.

VOL. IV.

FROM JUNE TO DECEMBER, 1817.

103676 447 63387 S.G.O. LIBRARY

*LONDON.*

PRINTED FOR THE PROPRIETORS,

BY WILLIAM THORNE, RED LION COURT, FLEET STREET.

To whom Communications, addressed to the Editors, are requested to be sent.

Sold by BALDWIN, CRADOCK, and JOY, Pater-Noster Row; HIGHLEY and SON, and T. & G. UNDERWOOD, Fleet Street; J. CALLOW, Crown Court, Soho; COX and SON, Borough; ANDERSON and CHASE, West Smithfield; W. BLACKWOOD, Edinburgh; J. CUMMING, Dublin; and may be had also by applying to the Clerks of the Roads, at the General Post Office.

### CORRESPONDENCE.

The Observer (No. 1) having given rise to some remonstrances, and also having been reproached on account of its anonymous character, Dr. Johnson, one of the Editors, acknowledges himself the author of that Number.

Dr. Emanuel Lazzaretto's Case of Traumatic Tetanus successfully treated, is received, and will appear in our next.

Alibert's Anatomical and Chemical Examinations of Dermoid Diseases, are received for the Miscellanies, together with some other papers for that department.

Dr. Felix's remarkable train of Conversions of Disease, will shortly appear.

Spurzheim on the deranged Manifestations of the Mind, and Mr. Marshall's Remarks on Arsenic, considered as a Poison and a Medicine, have been received, and will be reviewed in due course.

---

☞ Authors and Publishers are requested to send Copies of new Works for Review as early as possible after their publication. They will be reviewed early in proportion.

